# Sex differences in cognitive function among patients with bipolar disorder

**DOI:** 10.3389/fpubh.2025.1639276

**Published:** 2026-01-22

**Authors:** Zhonggang Wang, Gong Zhang, Haining Song, Zhenzhen Yang, Chen Wei, Yuying Cao

**Affiliations:** 1 Department of Psychiatry, Shandong Daizhuang Hospital, Jining, Shandong, China; 2 Department of Psychiatry, Zibo Mental Health Center, Zibo, Shandong, China; 3 Department of Medical Imaging, Affiliated Hospital of Jining Medical University, Jining, Shandong, China

**Keywords:** sex differences, cognition impairments, bipolar disorder, attention, executive function

## Abstract

**Background:**

Bipolar disorder (BD) is a severe mental illness characterized by high recurrence rates and notable cognitive deficits. Although sex differences are known to influence the clinical characteristics of BD, their specific impact on cognitive function remains unclear. This study aimed to examine the role of sex in shaping cognitive profiles within a large sample of BD patients.

**Methods:**

The sample included 243 individuals, 168 patients with first-diagnosed bipolar disorder (107 males and 61 females) and 75 healthy controls (44 males and 31females). Cognitive functions were assessed using the Continuous Performance Test (CPT), the Digital Span Test (DST), and the Wisconsin Card Sorting Test (WCST). Cognitive performance was compared between patient groups and healthy control groups.

**Results:**

Compared with female patients, the total scores of PPE and DST in male patients were significantly higher in the BD group. There were significant main effects of group on CPT, DST, and WCST. There were significant main effects of group and sex for total administered (TA) and non-perseverative errors (NPE) (all with *p-*value of <0.05). The TA and NPE of male patients are significantly higher than female patients. Among male subjects, the TA and NPE scores of BD patients were significantly higher than healthy controls. Among female subjects, the NPE score of BP patients was significantly higher than healthy controls. The hospital admission frequency was significantly and negatively correlated with attention function scores.

**Conclusion:**

Both male and female patients with BD exhibit cognitive impairment. Sex appears to play a crucial role in cognitive function among first-diagnosed patients with bipolar disorder. Male patients may be more vulnerable to deficits in cognitive transferability and attention stability, whereas female patients may be more susceptible to memory impairments.

## 1 Introduction

Bipolar disorder (BD), also known as manic-depressive illness, is a chronic, relapsing mental health condition typically characterized by recurrent episodes of depression and mania or hypomania (1). BD is a severe mental disease with high recurrence rates and marked cognitive impairment (2). Although sex differences are known to influence the clinical characteristics of BD (3), their specific impact on cognitive impairment remains a critical area of investigation.

For instance, while BD is equally prevalent in males and females, bipolar II disorder is more frequently diagnosed in females, who also exhibit an increased risk of rapid cycling and mixed episodes (4). Clinically, males often present with manic episodes and comorbid substance abuse, whereas females more commonly present with depressive episodes. Females may also show seasonal affective disorder episode patterns, atypical depressive symptoms (e.g., hypersomnia and weight gain), and higher rates of medical comorbidities, such as hypothyroidism, migraine, and obesity (5), although they are often associated with better medication adherence (6).

Beyond these clinical differences, cognitive dysfunction is a core feature of BD (7, 8). Cognitive impairments of BD patients may lead to poorer course and prognosis, and these impairments are possibly related to sex; however, how sex modulates these cognitive profiles remains a topic of debate. Cognitive dysfunction in BD patients demonstrates neurocognitive variants. Navarra-Ventura (9) reported that BD patients exhibit deficits in emotion recognition, affective Theory of Mind, and second-order cognitive Theory of Mind, including verbal learning, attention, immediate memory, and delayed memory (10, 11). Xu et al. (12) found that cognitive deficits in male BD patients showed poor attention and delayed memory, whereas other studies indicate that females generally outperform males in domains like language learning and memory (13), thereby creating an unclear picture of sex-specific vulnerabilities.

In conclusion, cognitive impairment is a key feature of BD that includes attention, memory, and executive functioning. These impairments influence recovery by preventing the return to optimal socio-occupational functioning and reducing quality of life. Thus, cognitive impairment is a therapeutic target for early intervention (14, 15). While previous research (11, 16) has confirmed that sex differences in the clinical course of BD, the impact of sex on cognitive functions among first-diagnosed BD patients remains incompletely understood. Therefore, this study aims to explore these sex-specific differences in cognitive functions among individuals recently diagnosed with BD.

## 2 Participants and methods

### 2.1 Participants

#### 2.1.1 Patient group

A total of 168 BD inpatients (107 male and 61 female patients) were recruited from Shandong Daizhuang Hospital in China between 1 July 2022 and 30 June 2023.

Inclusion criteria: (1) primary diagnosis meeting BD diagnostic criteria based on International Classification of Diseases-10 (ICD-10), confirmed independently by two experienced psychiatrists through structured clinical interviews and a review of medical records; (2) the Hamilton Depression Scale (HAMD) (24-item version) ≤ 7 points for a minimum of 2 weeks; (3) the Bech-Rafaelsen Mania Rating Scale (BRMS) ≤ 5 points for a minimum of 2 weeks; (4) age 18–60 years old; (5) no restrictions on sex; (6) at least having junior school education to understand the study questionnaire; and (7) Han ethnicity, residing in China.

Exclusion criteria: (1) diagnosis consistent with other psychiatric disorders according to the ICD-10, (2) patients with severe somatic illnesses, (3) receipt of electroconvulsive therapy within 2 weeks, (4) alcohol and other substance abuse, (5) history of traumatic brain injury and intelligence quotient (IQ) <70 points, and (6) disagree to participate in this study.

All enrolled patients were divided into two groups according to sex differences. A total of 168 patients with first-diagnosed BD were divided into the male group (*n* = 107) and the female group (*n* = 61). All patients in this cohort were treated with sodium valproate in combination with olanzapine; antidepressant treatment was needed during depression episodes. All drug dosages were systematically recorded.

#### 2.1.2 Control group

The healthy control (HC) group included staff, students, or registrars of the hospital during the same period, with sex and age matched to the BD patient group. A total of 75 healthy controls were enrolled including 44 males and 31 females, aged between 18–55 years, and mean of 25.31 ± 4.239 years.

All controls were screened to exclude any personal or first-degree family history of major psychiatric disorders.

### 2.2 Research method

All participants completed a comprehensive neuropsychological battery and provided detailed demographic and clinical information.

#### 2.2.1 Information collection questionnaire

A structured questionnaire was used to collect demographic and clinical data, including sex, age, occupation, family history, marital status, education level, age of onset, number of hospitalizations, and clinical scale scores (HAMD, BRMS).

The study protocol was reviewed and approved by the Ethics Committee.

#### 2.2.2 Assessments of mental condition

1. The Bech-Rafaelsen Mania Rating Scale (BRMS) (17).
  The BRMS mainly assesses the severity of the subject’s manic symptoms. A score of 0 represents no symptoms or a level similar to the subject’s normal level, 1 represents mild symptoms, 2 represents moderate symptoms, 3 represents significant symptoms, and 4 represents severe symptoms. Thus, the range of the total score is 0 to 44. The total score reflects the severity of the disease. The higher the total score, the more serious the disease. Score of 0–4 is no obvious manic symptoms, 6–10 is mild manic symptoms, and ≧22 is severe manic symptoms.
2. The Hamilton Depression Scale (24-item version) (HAMD-24) (18).
  The HAMD is used to assess depressive symptoms in patients with psychiatric disorders. There are three versions of this scale, including 17-item, 21-item, and 24-item versions. The 24-item version was used in this study, and the 24 items were scored using a five-point scale from 0 to 4, where0-none, 1-mild, 2-moderate, 3-severe, and 4-very severe. Total score <8: normal; total score from 8 to 20: possible depression; total score from 20 to 35: definite depression; and total score ≧35: severe depression.
3. The Positive and Negative Syndrome Scale (PANSS) (19, 20).
  The PANSS is mainly used to assess the presence or absence of psychiatric symptoms and the severity of each symptom in patients with psychiatric disorders. This scale was used in this study to assess the presence or absence of psychotic symptoms of the enrolled patients. Item scoring criteria: Each item of PANSS has a clear definition and a clear 7-point scale, and the 7-point scales are: 1-none; 2-very mild; 3-mild; 4-moderate; 5-moderate–severe; 6-severe; and 7-very severe.

### 2.3 Cognitive measurement instruments

1. The Wisconsin Card Sorting Test (WCST) (21–23).
  The WCST is a neuropsychological test that reflects subjects’ cognitive functions such as neuropsychological processes, generalization, working memory, cognitive transfer, information extraction, attention, categorization switching, categorization maintenance, stimulus recognition and processing, sensory input, and motor output. The test consists of 4 stimulus cards and 128 response cards. The WCST is suitable for measuring the cognitive function in patients.
  Measure indicators of the WCST: Trials Administered (TA), Total Correct Responses (CR), Percent Correct Responses (PCR), Total Errors (TE), Percent Errors (PE), Perseverative Responses (PR), Percent Perseverative Responses (PPR), Perseverative Errors (PE), Percent Perseverative Errors (PPE), Non-perseverative Errors (NPE), Percent Non-perseverative Errors (PNPE), Conceptual Level Responses (CLR), Percent Conceptual Level Responses (PCLR), Categories Completed (CC), Trials to Compete First Category (TCFC), Failure to Maintain Set (FM), Learning to Learn (L-L).
2. Measurements of Digit span test (DST) (24, 25).
  The DST mainly measures subjects’ short-term memory capacity and attention. The DST consists of two parts, Digit Span-Sequential Order (DS-SO) and Digit Span-Reversed Order (DS-RO). The DS-SO mainly reflects the memory ability and attention ability of the subjects; the digit span reversal mainly reflects the executive functions of the subjects, especially working memory and cognitive flexibility.
3. Measurements of the continuous performance test–identical pairs (CPT-IP) (26, 27).
  Several versions of CPT have been developed, and variations on the basic task have been used in research and clinical work. In a common format, the CPT presents several series of single visual stimuli (usually letters) displayed at brief intervals. In the CPT, subjects are required to respond when a letter is preceded by a designated target letter. Participants are asked to press the appropriate key for any letter presented. Multiple trials of different interstimulus intervals are presented, and scoring includes reaction times, number correct, and errors of omission and commission. In this study, the CPT-IP was used to measure the sustained attention function in BD patients and HC. The CPT is a performance/task-based assessment that evaluates multiple domains of attention, such as sustained attention, inattentiveness, impulsivity, and vigilance. It provides objective information regarding an individual’s attentional difficulties. Clients are presented with repetitive boring tasks and must maintain their focus over a period of time in order to respond to targets or inhibit response to non-targets.

### 2.4 Research flowchart

**Figure 1:**
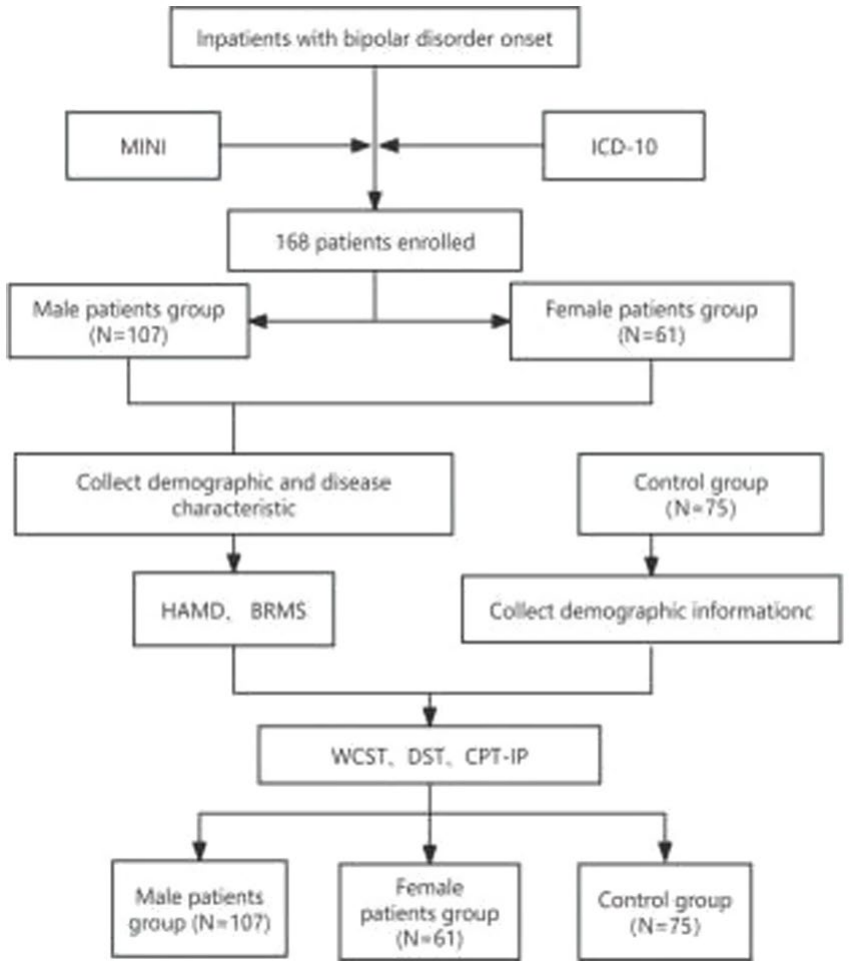
Research flowchart.

The flowchart illustrates the participants recruitment process, the diagnostic assessment tools, the grouping status, the total number of enrolled patients, and the number of cases in each groups. The details were showed in flowchart (Figure 1).

### 2.5 Statistical analysis

All data were statistically analyzed using IBM SPSS version 20.0. Measurement data such as age, age at first presentation, and other factors that conformed to normal distribution, they were expressed as (mean ± standard deviation), and one-way ANOVA was used for comparison between groups; those that did not conform to normal distribution were expressed as median (minimum, maximum), and non-parametric test (Mann–Whitney *U* test) was used. The chi-square test was used for sex, occupation, and other count data. The *χ*^2^ test was used to compare the differences in sex, occupation, family history, whether or not there were psychotic symptoms, whether or not there was a history of mental stimulation, and marital status among the first manic group, first depressive group, and normal control group. The one-way ANOVA was used to evaluate the differences in age and years of education among the first manic group, first depressive group, and normal control group. Independent sample *t*-test was used to compare the differences in the first onset of mania and first depressive group. Non-parametric test was used to calculate the differences in cognitive function scores among the first manic group, first depressive group, and normal control group. Spearman’s correlation analysis was performed to examine association between cognitive function impairment and age at onset, years of education, and number of hospitalizations in the first manic group and first depressive group. All analyzing data were conducted using raw scores (test level *α* = 0.05 with a two-sided test).

## 3 Results

### 3.1 Demographic characteristics of study participants

A higher proportion of male and female subjects in the BD and control groups reported stable occupations (*p* < 0.05 for all comparisons). There were no significant differences in demographic and clinical characteristics between male and female patients within the BD group. The detailed results are shown in Table 1.

**Table 1:** Sex differences in clinical characteristics and cognitive functioning among study participants.

Index		BD group				HC group			
		Male (*n* = 107)	Female (*n* = 61)	*X*^2^/*Z*	*p*	Male (*n* = 44)	Female (*n* = 31)	*X*^2^/*Z*	*p*
Age [years]		27.1 ± 0.6	27.5 ± 1.1	−0.876	0.381	24.8 ± 0.5	26.1 ± 0.9	−0.698	0.485
Education level [years]		11.3 ± 0.3	11.6 ± 0.5	−0.638	0.524	13.5 ± 0.5	14.3 ± 0.7	−0.864	0.387
Onset age [years]		23.2 ± 0.5	22.7 ± 0.7	−1.500	0.134	–	–	–	–
Admission times		3.8 ± 0.3	2.9 ± 0.3	−0.438	0.662	–	–	–	–
HAMD score		2.4 ± 0.1	2.5 ± 0.2	−0.421	0.674	–	–	–	–
BRMS score		1.7 ± 0.1	1.6 ± 0.1	−0.159	0.874	–	–	–	–
Occupation [*n*, %]	Yes	75 (70.1%)	30 (49.2%)	7.250	0.007	27 (61.4%)	29 (93.5%)	9.959	0.002
	No	32 (29.9%)	31 (50.8%)			17(38.6%)	2(6.5%)		
Marriage [*n*, %]	Unmarried	39 (36.4%)	21 (34.4%)	0.197	0.906	19 (43.2%)	12 (38.7%)	0.337	0.845
	Married	56 (52.3%)	34 (55.7%)			21 (47.7%)	15 (48.4%)		
	Divorce	12 (11.2%)	6(9.8%)			4 (9.1%)	4 (12.9%)		
Psychiatric family history [*n*, %]	Yes	35 (32.7%)	19 (31.1%)	0.044	0.835	–	–	–	–
	No	72 (67.3%)	42 (68.9%)			71 (100%)	36 (100%)		
Psychotic symptoms [*n*, %]	Yes	32 (29.9%)	25 (41.0%)	2.127	0.145	–	–	–	–
	No	75 (70.1%)	36 (59.0%)			–	–		
Suspected precipitating event for first episode [*n*, %]	Yes	30 (28.0%)	16 (26.2%)	0.064	0.800	–	–	–	–
	No	77 (72.0%)	45 (73.8%)			–	–		

BD, Bipolar disorder; HC, Health control.

### 3.2 Sex differences in cognitive function between two groups

(1) Within the HC group, female participants had significantly higher TE and NPE scores than male participants (*p* < 0.05). (2) Within the BD group, male patients exhibited significantly higher percent perseverative errors (PPE) and the total Digital Span Test (DST-Total) scores related to female patients (*p* < 0.05), indicating greater executive dysfunction (higher PPE) despite a stronger immediate memory span (higher DST-Total) (see Table 2).

**Table 2:** Sex differences in cognitive function between BD group and HC group.

Index		BD group		*Z*	*p*	HC group		*Z*	*p*
		Male	Female			Male	Female		
CPT	CPT2	2.5 ± 0.8	2.3 ± 1.1	−0.719	0.472	3.5 ± 0.8	3.4 ± 0.8	−0.267	0.79
	CPT3	1.7 ± 0.8	1.6 ± 0.9	−0.185	0.854	2.8 ± 0.8	3.0 ± 0.9	−0.679	0.497
	CPT4	0.9 ± 0.7	0.7 ± 0.7	−1.52	0.129	1.8 ± 0.8	1.9 ± 0.9	−0.49	0.624
WCST	RA	116.2 ± 16.9	109.7 ± 18.7	−1.769	0.077	100.4 ± 20.7	105.2 ± 16.7	−0.979	0.328
	CC	4.6 ± 1.5	4.7 ± 1.8	−0.656	0.512	5.5 ± 1.0	5.6 ± 1.0	−0.686	0.493
	TA	73.9 ± 69.2	66.6 ± 11.7	−1.534	0.125	74.1 ± 11.32	71.8 ± 16.6	−0.501	0.617
	CC	49.4 ± 23.7	43.1 ± 22.1	−0.198	0.843	6.0 ± 13.475.	30.9 ± 16.5	−1.356	0.175
	CR	59.1 ± 16.3	63.6 ± 14.8	−0.016	0.987	3 ± 9.0	71.7 ± 11.4	−1.528	0.127
	TE	20.1 ± 9.3	18.3 ± 14.2	−1.431	0.152	16.2 ± 8.5	18.0 ± 6.8	−1.985	0.047
	PCR	53.7 ± 19.4	57.2 ± 19.9	−0.218	0.828	71.7 ± 11.21	67.8 ± 12.5	−1.41	0.159
	TCFC	30.4 ± 20.3	25.8 ± 19.9	−0.613	0.54	2.3 ± 9.5	16.2 ± 17.1	−0.754	0.451
	PCLR	6.9 ± 6.5	8.2 ± 11.5	−0.809	0.418	5.5 ± 5.3	7.3 ± 8.0	−0.737	0.461
	PR	5.9 ± 5.3	7.0 ± 9.0	−0.465	0.642	5.1 ± 4.7	6.4 ± 6.6	−0.615	0.539
	PRE	42.4 ± 22.5	33.7 ± 21.3	−0.373	0.709	20.6 ± 11.61.	23.6 ± 12.5	−1.33	0.183
	PPE	1.5 ± 1.5	0.8 ± 1.0	−3.317	0.001	5 ± 2.0	1.2 ± 1.4	−0.056	0.955
	NPE	−8.4 ± 17.3	−4.0 ± 14.9	−1.772	0.076	1.2 ± 3.3	2.1 ± 12.2	−2.414	0.016
DST	SO	7.3 ± 1.0	7.2 ± 1.1	−1.658	0.097	7.9 ± 0.4	7.7 ± 0.4	−1.862	0.063
	RO	3.6 ± 1.1	3.2 ± 1.4	−0.993	0.32	4.8 ± 1.1	4.9 ± 1.2	−0.006	0.996
	Total	10.9 ± 1.7	10.4 ± 2.0	−6.354	<0.001	12.7 ± 1.3	12.6 ± 1.5	−0.466	0.641

BD, Bipolar disorder; HC, Healthy controls.

### 3.3 Main effects and interactions of neurocognitive variables

There were significant main effects of group for CPT, DST, and WCST (TA, CC, TE, PCR, PCLR, PR, NPE, L-L) (all *p* < 0.05). CPT, WCST (CC, TE, PCR, PCLR, L-L) of the BD group were significantly lower than the HC group. TA, WCST (TE, PR, NPE) of the BD group were significantly higher than HC group.

There were significant main effects of group and sex for TA and NPE, indicating there was an interaction between group and sex (all *p* < 0.05). The group affected TA and NPE scores of male patients; however, the TA and NPE scores of male patients were significantly higher than female patients (all *p* < 0.05). The BD group affected the NPE scores of female patients. The sex affected the TA and NPE scores of BD patients (see Table 3).

**Table 3:** Effects of group and sex on cognitive function of BD group and the healthy control group.

Cognitive functions		BD group		HC group		Group		Sex		Group × Sex	
		Male (*N* = 107)	Female (*N* = 61)	Male (*N* = 44)	Female (*N* = 31)	*F*	*p*	*F*	*p*	*F*	*p*
CPT	CPT2	2.5 ± 0.8	2.3 ± 1.1	3.5 ± 0.8	3.4 ± 0.8	69.440	<0.001	1.947	0.164	0.836	0.362
	CPT3	1.7 ± 0.8	1.6 ± 0.9	2.8 ± 0.8	3.0 ± 0.9	94.738	<0.001	0.009	0.926	0.890	0.346
	CPT4	0.9 ± 0.7	0.7 ± 0.7	1.8 ± 0.8	1.9 ± 0.9	92.837	<0.001	0.515	0.474	1.646	0.201
WCST	TA	116.2 ± 16.9	109.7 ± 18.7	100.4 ± 20.7	105.2 ± 16.7	14.876	<0.001	0.101	0.751	4.559	0.034
	CC	4.6 ± 1.5	4.7 ± 1.8	5.5 ± 1.0	5.6 ± 1.0	19.751	<0.001	0.202	0.654	0.019	0.890
	CR	73.9 ± 69.2	66.6 ± 11.7	74.1 ± 11.3	71.8 ± 16.6	0.174	0.677	0.522	0.471	0.144	0.705
	TE	49.4 ± 23.7	43.1 ± 22.1	26.0 ± 13.4	30.9 ± 16.5	34.910	<0.001	0.058	0.811	3.455	0.064
	PCR	59.1 ± 16.3	63.6 ± 14.8	75.3 ± 9.0	71.7 ± 11.4	35.608	<0.001	0.044	0.834	3.832	0.052
	TCFC	20.1 ± 9.3	18.3 ± 14.2	16.2 ± 8.5	18.0 ± 6.8	2.115	0.147	0.002	0.969	1.372	0.243
	PCLR	53.7 ± 19.4	57.2 ± 19.9	71.7 ± 11.2	67.8 ± 12.5	32.317	<0.001	0.007	0.936	2.138	0.145
	PR	30.4 ± 20.3	25.8 ± 19.9	12.3 ± 9.5	16.2 ± 17.1	27.700	<0.001	0.019	0.890	2.598	0.108
	PRE	6.9 ± 6.5	8.2 ± 11.5	5.5 ± 5.3	7.3 ± 8.0	1.087	0.298	1.741	0.188	0.049	0.825
	PPE	5.9 ± 5.3	7.0 ± 9.0	5.1 ± 4.7	6.4 ± 6.6	0.562	0.454	1.820	0.179	0.010	0.921
	NPE	42.4 ± 22.5	33.7 ± 21.3	20.6 ± 11.6	23.6 ± 12.5	32.538	<0.001	1.043	0.308	4.478	0.035
	FM	1.5 ± 1.5	0.8 ± 1.0	1.5 ± 2.0	1.2 ± 1.4	0.445	0.505	3.916	0.049	0.713	0.400
	L-L	−8.4 ± 17.3	−4.0 ± 14.9	−1.2 ± 3.3	2.1 ± 12.2	10.399	0.001	3.409	0.066	0.080	0.777
DST	SO	7.3 ± 1.0	7.2 ± 1.1	7.9 ± 0.4	7.7 ± 0.4	21.814	<0.001	0.787	0.376	0.080	0.778
	RO	3.6 ± 1.1	3.2 ± 1.4	4.8 ± 1.1	4.9 ± 1.2	66.686	<0.001	0.817	0.367	1.456	0.229
	Total	10.9 ± 1.7	10.4 ± 2.0	12.7 ± 1.3	12.6 ± 1.5	67.799	<0.001	1.153	0.284	0.487	0.486

### 3.4 Simple effect analysis: pairwise comparison–effects of sex and group on TA and NPE

#### 3.4.1 Effects of sex on TA and NPE

Within the BD group, male patients had significantly higher TA and NPE scores than females (*p* < 0.05). These higher scores reflect lesser performance, as they required more trials and made more non-perseverative errors. No significant difference was found between sexes in the HC group (*p* > 0.05). All details were shown in Table 4.

**Table 4:** Simple effect analysis: pairwise comparison—effects of sex on TA and NPE.

Groups	Sex	Mean deviation (I–J)	Standard error	Value^b^	95% Confidence	Interval for difference^b^
					Lower-bound	Upper-bound
Independent variable: TA						
BD group	Male–Female	6.429^a^	2.846	0.025	0.822	12.035
	Female–Male	−6.429^a^	2.846	0.025	−12.035	−0.822
Control group	Male–Female	−4.784	4.16	0.251	−12.979	3.41
	Female–Male	4.784	4.16	0.251	−3.41	12.979
Independent variable: NPE						
BD group	Male–Female	7.840^a^	3.54	0.028	0.867	14.813
	Female–Male	−7.840^a^	3.54	0.028	−14.813	−0.867
Control group	Male–Female	−3.054	5.174	0.556	−13.246	7.138
	Female–Male	3.054	5.174	0.556	−7.138	13.246

a The significance level of the mean difference was 0.05.

b Multiple comparison regulation, Bonfreni method.

#### 3.4.2 Effects of group on TA and NPE

For male subjects, the TA and NPE score of BD patients were significantly higher than HC subjects. For female subjects, the NPE score of BD patients was significantly higher than HC subjects. It suggested the attention of BD patients was less concentrated than the HC group. All details were shown in Table 5.

**Table 5:** Simple effect analysis: pairwise comparison—effects of group on TA and NPE.

Sex	Group	Mean deviation (I–J)	Standard error	p-value^b^	95% Confidence	Interval for difference^b^
					Lower-bound	Upper-bound
Independent variable: TA						
Male	BD group vs. Control group	17.544^a^	3.177	≦0.001	11.286	23.803
	Control group vs. BD group	−17.544^a^	3.177	≦0.001	−23.803	−11.286
Female	BD group vs. Control group	6.331	3.913	0.107	−1.377	14.039
	Control group vs. BD group	−6.331	3.913	0.107	−14.039	1.377
Independent variable: NPE						
Male	BD group vs. Control group	26.708^a^	3.951	≦0.001	18.924	34.492
	Control group vs. BD group	−26.708^a^	3.951	0.001	−34.492	−18.924
Female	BD group vs. Control group	15.814^a^	4.867	0.001	6.227	25.401
	Control group vs. BD group	−15.814^a^	4.867	0.001	−25.401	−6.227

a The significance level of the mean difference was 0.05.

b Multiple comparison regulation, Bonfreni method.

### 3.5 Correlation analysis of cognitive function scores in BD group

In BD group, the number of hospital admissions was significantly negatively correlated with CPT2 scores. BRMS score was significantly positively correlated with CC, CR, PCR, PCLR, and DST-RO, while BRMS score was significantly negatively correlated with TE and NPE (see Table 6).

**Table 6:** Correlation analysis of cognitive function score and affecting factors in BD group.

Influencing factors	CPT2	CPT3	CPT4	TA	CC	CR	TE	PCR	TCFC	PCLR	PR	PRE	PPE	NPE	FM	L-L	DST-SO	DST-RO	DST-Total
Onset age	*r*	−0.012	0.002	−0.006	−0.014	−0.031	−0.038	−0.056	0.033	−0.057	0.054	−0.062	−0.088	−0.067	−0.005	0.045	−0.036	0.14	0.043	0.106
	Sig (2-tailed)	0.878	0.977	0.938	0.855	0.689	0.629	0.469	0.672	0.464	0.488	0.421	0.259	0.385	0.948	0.563	0.667	0.07	0.579	0.173
Admission times	*r*	−0.233**	−0.139	−0.067	0.039	−0.061	0.035	−0.017	0.024	0.103	0.042	0.012	0.087	0.068	−0.058	0.102	−0.035	−0.085	−0.056	−0.07
	Sig (2-tailed)	0.002	0.072	0.385	0.613	0.435	0.653	0.827	0.76	0.189	0.586	0.878	0.261	0.384	0.452	0.189	0.668	0.274	0.47	0.368
Education level	*r*	0.001	−0.041	−0.097	0.086	−0.025	0.048	0.04	−0.06	0.084	−0.037	0.066	0.032	0.045	0.046	0.005	0.003	0.056	0.043	0.059
	Sig (2-tailed)	0.995	0.598	0.212	0.268	0.745	0.536	0.609	0.442	0.282	0.631	0.392	0.677	0.56	0.558	0.947	0.967	0.467	0.581	0.444
HAMD	*r*	0.02	0.026	0.062	−0.069	−0.025	0.033	−0.098	0.092	0.066	0.097	−0.089	−0.032	−0.028	−0.111	−0.076	0.103	0.08	−0.002	0.059
	Sig(2-tailed)	0.801	0.742	0.425	0.374	0.745	0.671	0.208	0.236	0.396	0.21	0.251	0.681	0.715	0.15	0.324	0.213	0.301	0.977	0.45
BRMS	*r*	0.071	0.141	0.084	−0.087	0.160*	0.200**	−0.175*	0.174*	0.028	0.185*	−0.147	−0.031	−0.012	−0.165*	0.106	0.158	−0.022	0.172*	0.102
	Sig (2-tailed)	0.361	0.069	0.281	0.261	0.038	0.009	0.023	0.024	0.725	0.017	0.057	0.694	0.875	0.033	0.17	0.054	0.778	0.026	0.188
Marital status	*r*	−0.012	0.003	−0.031	−0.011	−0.084	−0.138	0.003	0.007	−0.001	0.024	0.004	−0.016	0.007	−0.006	−0.027	−0.009	−0.04	0.088	0.051
	Sig (2-tailed)	0.879	0.973	0.693	0.884	0.278	0.075	0.972	0.93	0.994	0.76	0.954	0.834	0.928	0.943	0.727	0.912	0.611	0.255	0.508

## 4 Discussion

BD is a severe mental illness with high recurrence rates and neurocognitive impairments (28). Cognitive dysfunction is a typical characteristic of BD, and harms the quality of life of patients. However, the sex influences on cognitive impairment in BD remain unclear.

This study demonstrated that female HC exhibited poorer performance in cognitive transfer and attention sustainability compared to male HC. Male patients have worse cognitive transferability than female patients. The DST-total score of male patients is higher than that of female patients, which suggests that the memory span of male patients may be better than female patients. The results of this study are consistent with the findings of other researchers (1). This previous research (1) discussed the performance of 139 BD patients and 92 HCs using the Repeatable Battery for the Assessment of Neuropsychological Status (RBANS) scale and the Stroop color-word test. That study reported that male BD patients exhibited worse cognitive dysfunction than female patients in attention and delayed memory. Similarly, another study (29) found that BD patients demonstrated poorer attention performance compared with HCs. When compared with other mental disorders, BD was associated with poorer performance than unipolar depression but better performance than schizophrenia.

The TE score and PR score of BD patients was significantly higher than HC group, showing that the cognitive transfer ability of BD patients was impaired, especially in male patients. Zhong et al. (30) conducted a study using 92 BD patients and 43 HC. Executive function was assessed by WCST. The function of the left prefrontal cortex (PFC) in the BD patients was significantly lower than in HC. The NPE score of the BD group was higher than the HC group indicating that BD patients have difficulty with sustaining attention.

In this study, both group and sex had significant effects for TA and NPE, suggesting potential interactive effects. The Bonferroni multiple comparisons showed that sex and group had interactive effects on TA and NPE scores (all *p* < 0.05). Especially, the BD group affected the TA and NPE scores of male patients, and the attention retention ability of male patients was lower than that of the control group. Further, sex influenced TA and NPE scores of BD group patients. TA and NPE scores in the male BD patients were significantly higher than the females. However, the attention concentration ability of the female BD patients was superior the male patients. These findings highlight clear sex differences in the cognitive functions of BD patients. They suggest that sex-specific cognitive rehabilitation strategies may be needed, with emphasis on executive function training in males and memory support in females.

Other studies have reported similar findings regarding sex differences influencing the cognitive functions of BD patients. A study (31) included 462 individuals, comprising 347 patients with BD (148 males and 199 females) and 115 healthy controls (45 males and 70 females). Performance on a comprehensive neuropsychological battery assessing six cognitive domains and psychosocial functioning was compared between groups. The results indicated that male BD patients performed better than female patients in working memory, whereas female BD patients outperformed males in verbal learning and memory recognition tasks.

This study demonstrated a significant negative correlation between the number of hospitalizations and the CPT2 score. As hospitalizations increased, attentional impairments in patients with BD became progressively more severe. The greater the number of relapses and hospitalizations, the more advanced the disease condition and the more pronounced the associated cognitive function impairment (32). Therefore, optimizing treatment plan to prevent relapses is essential. Early intervention for cognitive impairment in BD patients is also critical for improving prognostic outcomes.

Future studies will investigate the neurobiological mechanisms underlying sex differences in cognitive functions of BD patients. For example, magnetic resonance imaging (MRI) will be employed to investigate potential sex-specific structural or functional brain differences. Such studies may help elucidate the neural substrates of cognitive deficits in BD and identify potential targets for therapeutic interventions. Moreover, longitudinal studies will be conducted to track the course of cognitive dysfunction in BD, with a focus on sex-specific trajectories. These studies will clarify how cognitive functions evolve over time and how they are influenced by treatment response, disease severity, and comorbidities.

## 5 Conclusion

The results of this study suggest that sex plays a major role in modulating the cognitive profile of first-diagnosed BD patients. Specifically, male patients appear more vulnerable to deficits in cognitive flexibility and attention stability, whereas female patients may be more susceptible to memory-related impairments. Recognizing these sex-specific vulnerabilities is essential for developing tailored interventions that enhance outcomes for individuals with BD. From a public health perspective, these findings highlight the importance of sex-sensitive clinical guidelines for the assessment and cognitive rehabilitation of patients with BD.

## 6 Limitations

This study has several limitations. First, its cross-sectional design preclude. This study did not conduct longitudinal follow-up observations to determine the trajectory of cognitive changes over time. Second, the sample was recruited from a single hospital in China, which may limit the generalizability of the findings to other ethnic and cultural groups. Third, all patients were receiving medication (sodium valproate and olanzapine) and the potential confounding effects of these drugs on cognitive performance cannot be fully disentangled from the effects of the illness itself. Finally, cognitive assessments were not conducted under blinded conditions, which could introduce bias. Future longitudinal studies with larger, more diverse, and drug-naïve samples are needed to validate these findings.

## Data Availability

The original contributions presented in the study are included in the article/supplementary material, further inquiries can be directed to the corresponding author.
